# Investigation on the applicability of a long-range reverse-transcription quantitative polymerase chain reaction assay for the rapid detection of active viruses

**DOI:** 10.1186/s12866-022-02723-7

**Published:** 2022-12-12

**Authors:** Masato Yasuura, Yuki Nakaya, Hiroki Ashiba, Takashi Fukuda

**Affiliations:** 1 Sensing System Research Center, National Institute of Advanced Industrial Science and Technology (AIST), Central 5, 1-1-1 Higashi, Tsukuba, Ibaraki, 305-8565 Japan; 2 Division of Virology, Department of Infection and Immunity, School of Medicine, Jichi Medical University, 3311-1 Yakushiji, Shimotsuke, Tochigi, 329-0498 Japan

**Keywords:** Infectivity, Inactivation, Heat-inactivation, Sodium hypochlorite, Ethanol

## Abstract

**Background:**

Although conventional polymerase chain reaction (PCR) methods are widely used in diagnosis, the titer of the pathogenic virus is difficult to determine based on the PCR. In our prior report, a long-range reverse-transcription quantitative PCR (LR-RT-qPCR) assay was developed to assess the titer of UV-irradiated influenza A virus (IAV) rapidly. In this research, we focused on whether the LR-RT-qPCR assay could evaluate the titer of IAV inactivated by other methods.

**Methods:**

IAV was inactivated by: heating at 100 °C for periods ranging from 1 to 15 min, treating with 0.12% sodium hypochlorite for periods ranging from 3 to 30 min, or treating with 70% ethanol for periods ranging from 10 to 30 min. Fifty percent tissue culture infectious dose (TCID_50_) assay was performed to confirm the efficacy of the inactivation methods, followed by LR-RT-qPCR to investigate the correlation between infectivity and copy number.

**Results:**

One minute heating, 3 min sodium hypochlorite treatment, or 10 min ethanol treatment was sufficient to deactivate IAV. Changes before and after the inactivations in the copy numbers on LR-RT-qPCR were significantly different among the inactivation methods. Heat-inactivation drastically decreased the copy number to below the cutoff value around 5 copies/μL after 5 min treatment. The inactivation time of heating estimated using LR-RT-qPCR was marginally higher than that determined using TCID_50_. However, the treatments with sodium hypochlorite or ethanol moderately and minimally affected the copy numbers obtained using LR-RT-qPCR (~ 1 digit or no copy number decrease), respectively.

**Conclusions:**

In addition to good applicability in UV-irradiation previously reported, the LR-RT-qPCR method is suitable for evaluating the effect of heat-inactivation on IAV infectivity. However, minor modifications may be made and investigated in the future to reduce the time intervals with TCID_50_. Although this method is not applicable for the ethanol inactivation, rapid evaluation of the effects of chlorination on IAV can be determined by comparing copy numbers before and after treatment using the LR-RT-qPCR method.

**Supplementary Information:**

The online version contains supplementary material available at 10.1186/s12866-022-02723-7.

## Background

Infection prophylaxis techniques are necessary to prevent the spread of emerging infectious diseases [1]. Monitoring the risks of viral infection in areas where cluster infections often occur, could be one of the methods for preventing the spread of infection. Sanitization of hands and equipment is considered as one of the important infectious disease control measures [1]. As current rapid virus measurement methods detect both viable and inactivated viruses, it is difficult to monitor the risk of viral infection directly. Based on these principles, the effect of sanitization needs to be evaluated. To monitor the infection risk, both reliability and rapidity are required. Various methods have been developed for virus detection [2–11]. Virus infectivity has been measured using cell culture-based infection tests such as plaque or focus assays because of their reliability [2–6]. However, these assays take several days to complete [6]. Polymerase chain reaction (PCR) is commonly used to detect viral genomes because of its rapidity and specificity [2, 4, 11]. However, conventional PCR is not capable of discriminating between infectious and non-infectious viruses as PCR detects the viral genome regardless of their infectivity. Long-range quantitative PCR (LR-qPCR) and long-range reverse-transcription quantitative PCR (LR-RT-qPCR) have been developed as methods to evaluate infectivity of both DNA and RNA viruses without cell culture [12–17]. In LR-RT-qPCR targeting RNA viruses, LR-RT is performed to prepare complementary DNA prior to qPCR [12, 14]. Subsequently qPCR occurs at a locus that is separate from the RT-priming site to reduce the effects of fragmented RNA. Fragmented viral RNAs are not included in the amplification. Therefore, the LR-RT-qPCR is more influenced by the damage of viral RNA than the conventional RT-qPCR. In a previous study [18], we developed an LR-RT-qPCR, which can determine the effects of UV-irradiation on influenza A virus (IAV) infectivity. The IAV genome consists of eight negative-sense, single-stranded RNA segments whose lengths are between 2.3 kb and 0.8 kb (A/Panama/2007/1999[H3N2], NCBI GenBank). All the RNA segments have highly conserved, short sequences at both the 5′- and 3′- termini, which are designed as universal primer pairs for the amplification of the IAV genome. In our LR-RT-qPCR for IAV, we used the 3′-terminus sequences as the priming site for the LR-RT reaction. The qPCR reaction was performed using specific primer pairs for each segment at the end of the 5′- terminus. The LR-RT-qPCR correlated highly with the infectivity of UV-irradiated IAV especially when targeting segment 3 of the IAV genome, which codes for polymerase acidic protein (PA). However, it was not determined if the LR-RT-qPCR for PA could predict the effects of other sanitization methods.

Enveloped viruses can be inactivated by several sanitization methods including heating, ethanol, and sodium hypochlorite treatments [19–23]. Heating sterilization is often performed by boiling a liquid. Sanitization using ethanol or sodium hypochlorite is often performed by sinking a tool in an aqueous solution or perfusing an aqueous solution into a tool. This study investigated whether the LR-RT-qPCR method [18] can confirm the effects of the above sanitization methods on the inactivation of IAV and to monitor viral infection risks of IAV, without cell culture-based infectivity assays. This study evaluated IAV as a representative of enveloped RNA viruses.

## Methods

### Cell culture and virus preparation

Madin–Darby canine kidney (MDCK) cells (accession no. CCL-34, ATCC, Manassas, VA) were maintained in Dulbecco’s Modified Eagle’s Medium (Fujifilm Wako Pure Chemical Corporation, Osaka, Japan) supplemented with 10% fetal bovine serum (Fujifilm Wako Pure Chemical Corporation), 100 U/mL penicillin, and 100 μg/mL streptomycin (Fujifilm Wako Pure Chemical Corporation) at 37 °C in a 5% CO_2_ atmosphere. IAVs (A/Panama/2007/1999 [H3N2] [Panama] and A/Puerto Rico/8/1934 [H1N1] [PR8]) were propagated in MDCK cells and the aliquots were stored as IAV stocks at ˗80 °C. The titer of the IAV stocks was determined using the 50% tissue culture infectious dose (TCID_50_) assay as described below.

### Disinfection of IAV

Heat-inactivation was performed by heating the 140 μL IAV stocks at 100 °C for 1, 3, 5, 7.5, 10, and 15 min or 1, 3, and 5 min for Panama or PR8 strains, respectively, using a dry block incubator (MyBL-100, As One Corporation, Osaka, Japan) [19]. After the heating, the heated IAV stocks were immediately put into an icebox to stop heating. The treated samples were used for the TCID_50_ assays or viral RNA isolation. The non-treated (0 min heated) samples (140 μL) were used for the TCID_50_ assays or viral RNA isolation without any special processing.

Sodium hypochlorite treatment was performed by incubating the mixtures of the 137.7 μL IAV stocks and 6% sodium hypochlorite (2.3 μL) for 10, 20, and 30 min or 3, 5, and 10 min for Panama or PR8 strains, respectively, according to published guidelines [20], then subsequently 10-fold diluted in phosphate-buffered saline (PBS) (−) (Fujifilm Wako Pure Chemical Corporation) to stop the treatment. The non-treated (0 min treated) samples (137.7 μL IAV stocks) were mixed with 2.3 μL PBS (−) and the mixtures were 10-fold diluted in PBS (−). The diluted samples were used for the TCID_50_ assays and viral RNA isolation.

For the ethanol treatment, the 42 μL IAV stocks were mixed with 100% ethanol (98 μL) and incubated for 10, 20, and 30 min [21], then 10-fold diluted in PBS (−) as performed in the sodium hypochlorite treatment. The non-treated (0 min treated) samples (42 μL IAV stocks) were mixed with 98 μL PBS (−) and the mixtures were 10-fold diluted in PBS (−). The diluted samples were used for the TCID_50_ assays and viral RNA isolation. The sodium hypochlorite treatment and ethanol treatment were performed at room temperature (approximately 20 °C) on a table. Regardless of the inactivation methods, three individual IAV suspensions per each time condition of inactivation were separately treated.

### TCID_50_ assay

IAV infectious titer was analyzed by TCID_50_ assay according to the published studies with minor modifications [2, 5]. Briefly, MDCK cells were split into 96-well culture plates at a concentration of 2 × 10^4^ cells/well. Regardless of the inactivation methods, a sample (non-treated or treated) was serially diluted 10-fold in the Eagle’s minimal essential medium (E-MEM) (Nissui Pharmaceutical Co., Ltd., Tokyo, Japan) supplemented with 1 μg/mL trypsin (Sigma-Aldrich Japan, Tokyo, Japan). MDCK cells were inoculated with 100 μL of each IAV dilution and incubated at 37 °C for 1 h. The inoculum was removed, and the cells were washed once with PBS to eliminate the unbound virus particles. The cells were overlaid with the E-MEM and incubated at 37 °C for a week. The ratio of wells where cytopathic effect (CPE) was observed and calculated by multiplying the relevant dilution factors to calculate the infectious titer. Infectious titers were calculated using the Behrens–Karber method [24] and defined as the log of TCID_50_ units [log (TCID_50_/0.1 mL)]. The data represents the mean ± standard error of the three independent experiments. The limit of detection (LoD) of the TCID_50_ assays was 0.75 log (TCID_50_/0.1 mL). For convenience, the values of stock where no CPE [lower than 0.50 log (TCID_50_/0.1 mL)] was observed were considered as zero [log (TCID_50_/0.1 mL)] and were included as data points in the figures.

### Long-range RT-qPCR

PA was analyzed using LR-RT-qPCR to evaluate the effects of inactivation treatments in this study [18]. Regardless of the inactivation methods, viral RNA was isolated from 140 μL sample (non-treated or treated) using the QIAamp Viral RNA Mini Kit (Qiagen K.K., Tokyo, Japan) according to the manufacturer’s instructions. Each RNA sample was eluted in 60 μL of elution buffer. The isolated RNA was subjected to reverse-transcription using SuperScript III (Thermo Fisher Scientific K.K., Tokyo, Japan) and an RT primer (5′-AGCGAAAGCAGG-3′) that binds specifically at the 3′ terminus of IAV PA [25]. The isolated RNA was then digested using RNase H included in SuperScript III. qPCR was then performed using Power SYBR Green PCR Master Mix (Thermo Fisher Scientific K.K.) according to the manufacturer’s instructions. Thermal cycling was performed on the LightCycler 96 (Roche Diagnostics K.K., Tokyo, Japan) under the following cycling conditions: activation at 95 °C for 10 min, and 40 cycles of PCR at 95 °C for 15 s and 60 °C for 1 min. The primer pair for the qPCR was Fw: 5′-GGATTTTCAGCGGAGTCAAG-3′ and Rev.: 5′-GGAGTTGAACCAAGACGCAT-3′ as used in the previous study [18]. DNA standard samples, measured the concentration by a spectrophotometer Nanodrop (Thermo Fisher Scientific K.K., Tokyo, Japan), were 10-fold serially diluted and subjected to the RT-qPCR analyses at the same time with the IAV samples to make standard curves. The IAV standard curve was created by measuring the Ct values of a serially diluted plasmid DNA containing the PCR amplicon [18]. Values below that of the most diluted standard were defined as “not detected” and included in the datasets as zero. Therefore, the LoD (cut-off copy number) of the LR-RT-qPCR assay for the PA segment was 5.53 copies/μL. Copy number quantification was performed using the IAV standard curves. Each copy number was divided by the copy number of non-treated IAV to calculate the copy number ratio. Data represent the three independent experiments and the mean ± standard error of them.

### Statistics

The significance of all the datasets in the TCID_50_ and LR-RT-qPCR assay was verified using the Kruskal–Wallis test. The Dunn–Bonferroni post hoc method was conducted to assess the significance between each pair of datasets of averaged technical replicates in the TCID_50_ and LR-RT-qPCR assay. Values considered to be significant had *P*-values < 0.05.

## Results

### Effect of heat treatment on IAV infectivity and LR-RT-qPCR assay

**Fig. 1:**
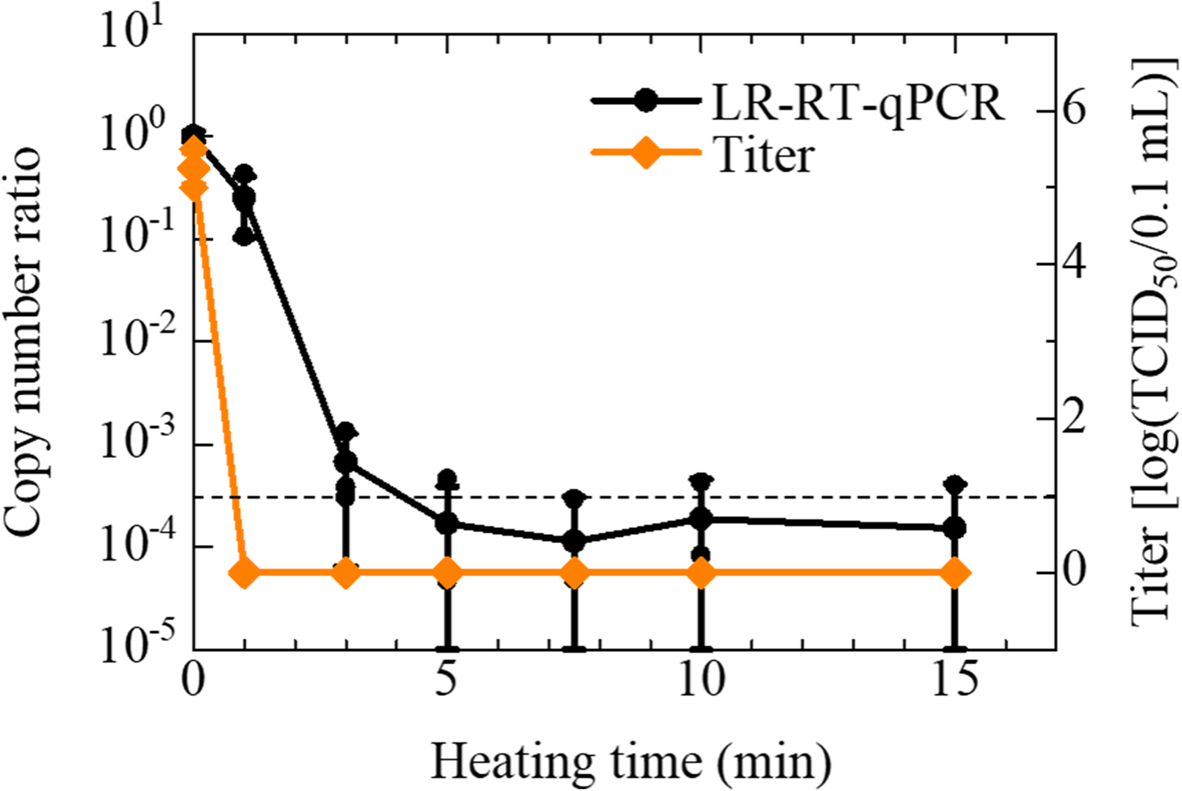
Effect of 100 °C heating on IAV (Panama strain) infectivity and copy numbers. IAV suspensions were heated with a block incubator for increasing periods of time between 1 and 15 min. IAV infectivity was measured by TCID_50_ assay using MDCK cells. The copy numbers were measured using LR-RT-qPCR targeting the PA segment. The titers are plotted on the graph and shown as log (TCID_50_/0.1 mL) (◆). Assays were performed independently three times with quadruplicate wells per dilution and values represent the mean ± standard error. No CPE was observed after 1 min of heating, and these were defined as 0 (means ‘not detected’) for convenience. The ratio of copy numbers obtained using LR-RT-qPCR is also plotted on the graph (●). Assays were carried out independently three times with triplicate wells per sample and plots represent the mean ± standard error and individual values. A broken line indicated the LoD of LR-RT-qPCR. The data indicated in Additional Table 1 (See ‘Additional Tables’)

We performed the heat-inactivation to the IAV stocks (Panama strain) to determine the correlation between the LR-RT-qPCR and the TCID_50_ assay. The heat-inactivation conditions were determined based on a previous study that showed heating at 100 °C for several minutes sterilizes most of the pathogenic viruses [19]. The TCID_50_ assay was conducted to establish the required time for complete inactivation of the IAV stock. As shown in Fig. 1, 1 min heating was sufficient to inactivate the IAV stock (Bonferroni-adjusted *P* = 0.014) whose original average titer, in this assay, was 5.25 log (TCID_50_/0.1 mL). LR-RT-qPCR was then performed to determine how the heat-inactivation affected the IAV genome (Fig. 1). The copy number ratio showed a 3-digits reduction on 3 min heating (Bonferroni-adjusted *P* = 0.111) and plateaued after 5 min heating (Bonferroni-adjusted *P* < 0.001), with approximately 25% of IAV genome being detectable on 1 min heating despite the IAV stock having lost infectivity. At the various time intervals, 5 to 15 min, PCR was negative in most of the wells (20/36, 56%) but still positive with a small copy number (< 5.53 copies/μL) in the other wells (16/36, 44%).

### Effect of sodium hypochlorite treatment on IAV infectivity and LR-RT-qPCR assay

**Fig. 2:**
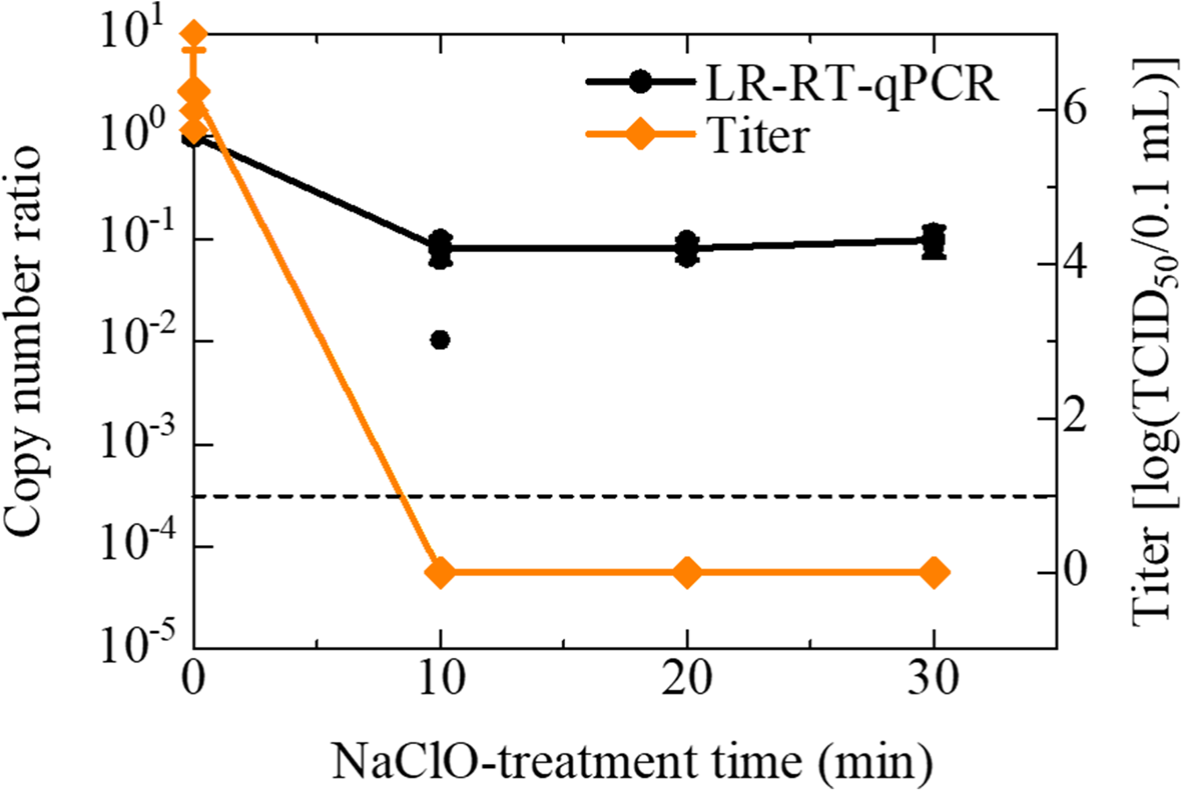
Effect of 0.12% sodium hypochlorite on IAV (Panama strain) titers and copy numbers. IAV suspensions were treated with 0.12% sodium hypochlorite for increasing periods between 10 and 30 min. The titer and copy numbers of treated IAV were determined by TCID_50_ assay using MDCK cells and by LR-RT-qPCR targeting the PA segment, respectively. The titers are plotted on the graph and shown as log (TCID_50_/0.1 mL) (◆). Assays were carried out independently three times with quadruplicate wells per dilution and values represent the mean ± standard error. After over 10 min treatment, there was no CPE in all conditions. They were defined as 0 (means ‘Not detected’) for convenience. The ratio of copy numbers obtained using LR-RT-qPCR is also plotted on the graph (●). Assays were performed with triplicate wells per sample and plots represent the mean ± standard error and individual values. A broken line indicated the LoD of LR-RT-qPCR. The data indicated in Additional Table 2 (See ‘Additional Tables’)

According to the guideline for disinfection and sterilization, issued by the Centers for Disease Control and Prevention (CDC) [20], over 0.1% hypochlorite sterilizes various bacteria and viruses. We therefore decided to use 0.12% sodium hypochlorite, as a concentration higher than 0.1%, to inactivate IAV in the experiment for sodium hypochlorite treatment. The margin of the concentration is a countermeasure for decreasing in concentration caused by the spontaneous decomposition of sodium hypochlorite. The infectivity of the IAV stock (Panama strain), original average titer of 6.25 [log (TCID_50_/0.1 mL)] in this assay, was reduced after 10 min sodium hypochlorite treatment (Bonferroni-adjusted *P* = 0.045) (Fig. 2). Although there was approximately 90% reduction in the copy number observed at 10 min (Bonferroni-adjusted *P* = 0.007), approximately 10% IAV genome was still detected at all the time intervals in the LR-RT-qPCR assay (Fig. 2).

### Effect of ethanol treatment on IAV infectivity and LR-RT-qPCR assay

**Fig. 3:**
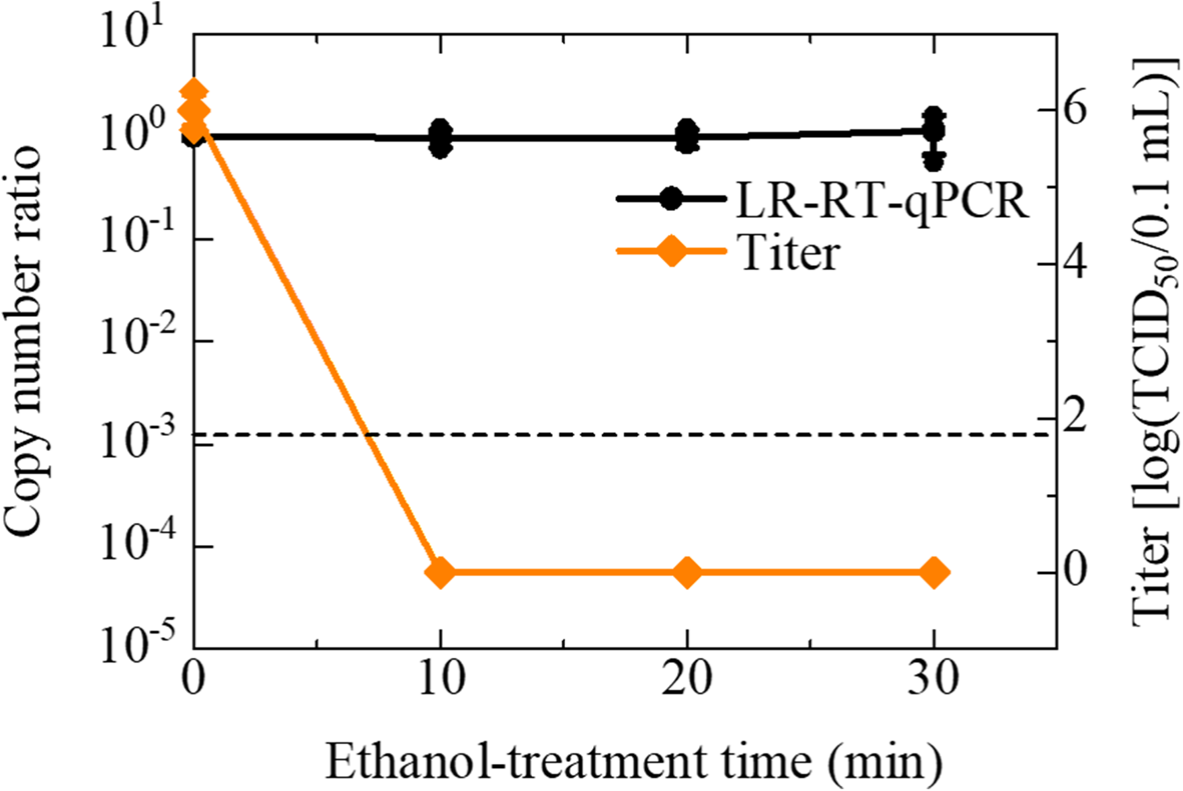
Effect of 70% ethanol on IAV (Panama strain) infectivity and copy numbers. IAV suspensions were treated with 70% ethanol for increasing periods between 10 and 30 min. The titers of treated IAV were measured by TCID_50_ assay using MDCK cells. The titers are plotted on the graph and shown as log (TCID_50_/0.1 mL) (◆). Assays were performed independently three times with quadruplicate wells per dilution and values represent the mean ± standard error. After over 10 min treatment, there was no CPE in all conditions. These titers were defined as 0 (means ‘Not detected’) for the convenience of plotting. The copy numbers were measured using LR-RT-qPCR targeting the PA segment. The ratio of copy numbers obtained using LR-RT-qPCR is also plotted on the graph (●). Assays were carried out independently three times with triplicate wells per sample and plots represent the mean ± standard error and individual values. A broken line indicated the LoD of LR-RT-qPCR. The data indicated in Additional Table 3 (See ‘Additional Tables’)

Conditions of ethanol treatment to the IAV stocks (Panama strain) were determined by the CDC’s guideline [20] and a previous study [21]. According to the references, 70% ethanol inactivates enveloped viruses. Similar to that observed for the sodium hypochlorite treatment, the infectivity of the original titer of 6 [log (TCID_50_/0.1 mL)] disappeared within 10 min (Bonferroni-adjusted *P* = 0.045) (Fig. 3). However, ethanol treatment had no effect on the copy number (*P* = 0.587) until after 30 min using the LR-RT-qPCR assay (Fig. 3).

### Additional experiments using PR8 strain

To confirm that these results were replicable in the other subtypes of IAV, PR8 strain was investigated in the heat-inactivation and sodium hypochlorite treatment. Three different lots of PR8 stocks were used for the analyses. The heated samples (heated for 0, 1, 3, and 5 min) and sodium hypochlorite-treated samples (treated for 0, 3, 5, and 10 min) were measured by the LR-RT-qPCR, conventional RT-qPCR, and TCID_50_ assay. The incubation times of heating and sodium hypochlorite treating were determined as the reduction of the copy number ratio of Panama strain plateaued after 5 min heating or 10 min sodium hypochlorite treatment.

Conditions of the conventional RT-qPCR, such as the probe and primer sequences and thermal cycling, were derived from the IAV detection protocols released by the World Health Organization (WHO) [26] and our previous report [18]. The same isolated RNA solution was used for the conventional RT-qPCR and LR-RT-qPCR. The isolated RNA was subjected to RT-qPCR with the TaqMan Fast Virus 1-Step Master Mix (Thermo Fisher Scientific K.K., Tokyo, Japan), a TaqMan probe (5′-FAM-ATYTCGGCTTTGAGGGGGCCTG-MGB-3′), and a primer pair (Forward: 5′-CCMAGGTCGAAACGTAYGTTCTCTCTATC-3′, Reverse: 5′-TGACAGRATYGGTCTTGTCTTTAGCCAYTCCA-3′). Thermal cycling was performed on the LightCycler 96 (Roche Diagnostics K.K., Tokyo, Japan) for reverse transcription at 50 °C for 5 min, denaturation of the RT polymerase at 95 °C for 20 s, and 45 cycles of PCR at 95 °C for 3 s and 60 °C for 30 s. Copy number quantification was carried out with the simultaneous measurement of the 10-fold serially diluted standard DNA. An assay was performed in a duplicate experiment. The LoD of the conventional RT-qPCR assay was 0.203 copies/μL.

**Fig. 4:**
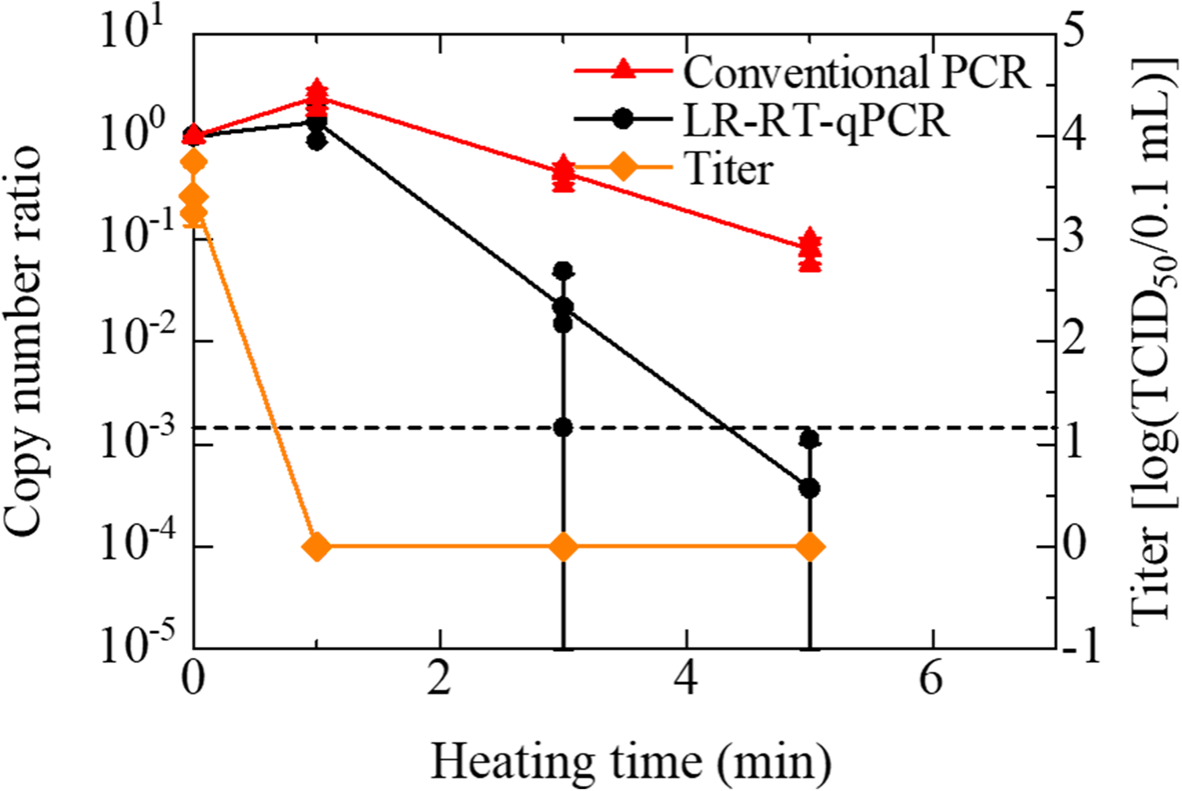
Effect of 100 °C heating on IAV (PR8 strain) infectivity and copy numbers. IAV suspensions were heated with a block incubator for increasing periods of time between 1 and 5 min. IAV infectivity was measured by TCID_50_ assay using MDCK cells. The copy numbers were measured using LR-RT-qPCR targeting the PA segment. The titers are plotted on the graph and shown as log (TCID_50_/0.1 mL) (◆). Assays were performed independently three times with quadruplicate wells per dilution and values represent the mean ± standard error. No CPE was observed after 1 min of heating, and these were defined as 0 (means ‘not detected’) for convenience. The ratios of copy numbers obtained using LR-RT-qPCR and conventional RT-qPCR are also plotted on the graph (● and ▲), respectively. Assays were carried out independently three times with three different lots of the PR8 stocks and plots represent the mean ± standard error and individual values. A broken line indicated the LoD of LR-RT-qPCR. The data indicated in Additional Table 4 (See ‘Additional Tables’)

**Fig. 5:**
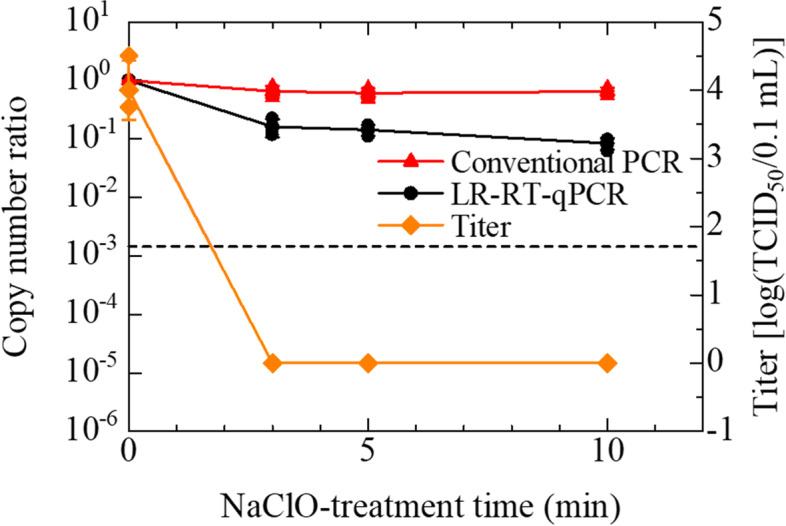
Effect of 0.12% sodium hypochlorite on IAV (PR8 strain) titers and copy numbers. IAV suspensions were treated with 0.12% sodium hypochlorite for increasing periods between 3 and 10 min. The titers of treated IAVs were determined by TCID_50_ assay using MDCK cells. The copy numbers of treated IAV were measured by LR-RT-qPCR targeting the PA segment and by conventional RT-qPCR targeting the M segment, respectively. The titers are plotted on the graph and shown as log (TCID_50_/0.1 mL) (◆). Assays were carried out independently three times with quadruplicate wells per dilution and values represent the mean ± standard error. After over 3 min treatment, there was no CPE in all conditions. They were defined as 0 (means ‘Not detected’) for convenience. The ratios of copy numbers obtained using LR-RT-qPCR and conventional RT-qPCR are also plotted on the graph (● and ▲), respectively. Assays were performed with three different lots of the PR8 stocks and plots represent the mean ± standard error and individual values. A broken line indicated the LoD of LR-RT-qPCR. The data indicated in Additional Table 5 (See ‘Additional Tables’)

In the heat-inactivation, the infectivity of the PR8 stocks, whose original average titer was 3.42 [log (TCID_50_/0.1 mL)] in this assay, was lost after 1 min heating at 100 °C (Fig. 4). The copy number ratio of the PR8 stocks on the LR-RT-qPCR showed a nearly 2-digits reduction in 3 min heating and the copy number reached below the LoD (4.97 copies/μL) after 5 min heating, which is consistent with Panama strain. On the other hand, the copy number ratio of the PR8 stocks on the conventional RT-qPCR showed an approximately 1-digit reduction in 5 min heating. In the sodium hypochlorite treatment, the infectivity of the PR8 stocks, whose original average titer was 4.0 [log (TCID_50_/0.1 mL)] in this assay, was lost after 3 min treatment by sodium hypochlorite (Fig. 5). The copy number ratio of the PR8 stocks on LR-RT-qPCR reduced 80–90% in 3–5 min treatment and more than 1-digit reduction in 10 min treatment. On the other hand, the copy number ratio of the PR8 stocks on the conventional RT-qPCR showed a 35–40% reduction in 3–10 min treatment.

## Discussion

It is desirable to evaluate infectious viruses by absolute values for a rapid virus measurement in infectious risk monitoring, i.e., to detect only active viruses. On the other hand, an assessment method confirming the effect of sanitization does not have to be based on the absolute values but is allowed to be just based on the relative values before and after the sanitization to evaluate changes in the infectious risks. Moreover, it is ideal in practical applications that these methods are not influenced by the causes of viral inactivation. Our previous study indicated that infectivity of UV-irradiated IAV can be evaluated using LR-RT-qPCR targeting the PA segment [18]. Thus in this paper, we examined whether our LR-RT-qPCR method can evaluate the effect of heat and chemicals on the infectivity of IAV.

**Table 1:** The prediction ability of IAV-infectivity using LR-RT-PCR targeting the PA segment

Treatment	Evaluation ability	Needed information
UV irradiation [18]	◎	Copy number after treatment only
Heating	◎	Copy number after treatment only *Over 5 min treatment and use of the cutoff value (= LoD) are required
Sodium hypochlorite	△	Copy numbers both before and after treatment *Copy number decrease: ~ 1 digit
Ethanol	✕	Cannot predict

The table indicates the ability of LR-RT-qPCR in assessing the infectivity of IAV and needed information for the evaluation. The evaluation ability is conveniently indicated by symbols

◎: The LR-RT-qPCR can detect infectious IAV to distinguish it from IAV inactivated by the treatment

△: The LR-RT-qPCR can be applied to confirm the effect of the sanitization

✕: The LR-RT-qPCR cannot evaluate infectivity of IAV inactivated by the treatment

Table 1 shows the ability of IAV-infectivity evaluation with LR-RT-PCR targeting the PA segment. The copy numbers of IAV (at least Panama and PR8 strains) on the LR-RT-qPCR can evaluate the infectivity of IAV heated at 100 °C by using the cutoff value (= LoD), similar to that of the UV-irradiated IAV [18]. Supplementally, compared with the copy numbers on the LR-RT-qPCR, the copy numbers on the conventional RT-qPCR measurements were not or weakly influenced by the heat-inactivation (See Fig. 4 and ‘Supplementary’). This indicates that LR-RT-qPCR can be applied to infectious risk monitoring at the site where there may be remnants of heat-inactivated IAVs, although a slight modification to shrink the time-gap with TCID_50_ is desired for practical applications. Approximately 3–4 digit decrease in the copy number of IAV (Figs. 1 and 4), including zero-copy in some wells, was probably caused by thermal denaturation of RNA, such as thermal scission of phosphodiester bonds and thermal denaturation of ribonucleoprotein [21, 27]. However, the loss of IAV infectivity by 1 min of heating (Figs. 1 and 4) might be caused by not only the RNA denaturation but also the denaturation of other proteins, for example, hemagglutinin and neuraminidase [21]. In fact, the 140 μL IAV stocks (8 HA/50 μL before heating) with heating 1 min or over 3 min showed hemagglutination negative (< 2 HA/50 μL) in the hemagglutination assay. Therefore, the infectivity of IAV heated for 1 min or 3 min was lost even though LR-RT-qPCR detected the IAV genomes of not negligible copy numbers.

In the case of the treatment with 0.12% sodium hypochlorite, the effect of the sanitization was evaluated by comparing copy numbers before and after the treatments (Table 1). This indicates that LR-RT-qPCR can be applied to confirm the effect of sanitization using sodium hypochlorite. The 90% decrease in the copy number of IAV on the LR-RT-qPCR (Figs. 2 and 5) was probably caused by RNA degradation by the strong oxidizing power of sodium hypochlorite [28, 29]. However, the loss of IAV infectivity after 10 min of treatment (Fig. 2) might be caused by not only the RNA degradation but also the denaturation of viral proteins [20]. In fact, the infectivity of IAV (PR8 strain) treated for 3 min was lost despite the copy number ratio of PR8 stocks on the LR-RT-qPCR and conventional RT-qPCR showed approximately 15 and 65% (Fig. 5), respectively.

In contrast, the infectivity of 70% ethanol-treated IAV cannot be evaluated using the LR-RT-qPCR (Table 1). No decrease in copy number of IAV was observed (Fig. 3), which is expected considering ethanol is often employed for RNA extractions [30]. The loss of IAV infectivity after 10 min of ethanol treatment (Fig. 3) could be caused by the destruction of the viral envelope and the denaturation of viral proteins [20, 21]. Therefore, the LR-RT-qPCR for the risk assessment of the infectivity of ethanol-treated viruses requires some additional improvement in future work.

Although our LR-RT-qPCR method currently has some limitations as a general-purpose infection risk monitoring method, the results in UV-irradiation [18] and heat inactivation indicate a potential application for risk monitoring. Furthermore, LR-RT-qPCR is effective for UV irradiation, heat inactivation, and sodium hypochlorite treatment as a method for confirming the effect of sanitization. At the present stage, the estimation of viral infectivity using both conventional methods, such as the HA assay, and the LR-RT-qPCR could be beneficial to improve the accuracy of the assessments. At least, a conventional method has to be introduced to evaluate the effects of ethanol sanitization. We would like to plan the improvement of the applicable sanitization methods and the verification of the applicability to clinical samples in further study.

## Conclusions

In summary, our study indicated that IAV infectivity can be evaluated by our LR-RT-qPCR method to determine the effect of heat-inactivation and sodium-hypochlorite-treatment. However, the LR-RT-qPCR cannot evaluate the IAV infectivity with ethanol as a treatment. Although the method suggested in this paper has limitations, it offers a new technique that is effective as a rapid evaluation of viral infectious risks.

## Supplementary Information

**Additional file 1: Supplementary Fig. 1.** Effect of 100 °C heating on IAV infectivity and copy numbers. Supplementary information describes the conventional RT-qPCR measurements of the heat-inactivated samples. The method and results of the performed conventional RT-qPCR assay are indicated in the file.

**Additional file 2: Additional Table 1.** Effect of 100 °C heating on IAV (Panama strain) infectivity and copy numbers. (Data of Fig. 1). **Additional Table 2.** Effect of 0.12% sodium hypochlorite on IAV (Panama strain) titers and copy numbers. (Data of Fig. 2). **Additional Table 3.** Effect of 70% ethanol on IAV (Panama strain) infectivity and copy numbers. (Data of Fig. 3). **Additional Table 4.** Effect of 100 °C heating on IAV (PR8 strain) infectivity and copy numbers. (Data of Fig. 4). **Additional Table 5.** Effect of 0.12% sodium hypochlorite on IAV (PR8 strain) titers and copy numbers. (Data of Fig. 5). **Additional Table 6.** Effect of 100 °C heating on IAV (Panama strain) copy numbers. (Data of Supplementary Fig. 1). Additional Tables. Supplementary information describes the data of all figures.

## Data Availability

All data generated or analyzed during this study are included in this published article.

## Abbreviations

PCR: Polymerase chain reaction
LR-RT-qPCR: Long-range reverse-transcription quantitative polymerase chain reaction
IAV: Influenza A virus
UV: Ultraviolet
PA: Polymerase acidic protein
MDCK: Madin–Darby canine kidney
TCID_50_: 50% tissue culture infectious dose
E-MEM: Eagle’s minimal essential medium
CPE: Cytopathic effect
CDC: Centers for Disease Control and Prevention
