# Supplementary material for: Investigation on the applicability of a long-range reverse-transcription quantitative polymerase chain reaction assay for the rapid detection of active viruses
Source: BMC Microbiol. 2022 Dec 12;22:300. doi: 10.1186/s12866-022-02723-7 (PMC9743722; doi:10.1186/s12866-022-02723-7)
Supplement: Supplementary file 1 — Additional file 1: Supplementary Fig. 1. Effect of 100 °C heating on IAV infectivity and copy numbers. Supplementary information describes the conventional RT-qPCR measurements of the heat-inactivated samples. The method and results of the performed conventional RT-qPCR assay are indicated in the file. [file 12866_2022_2723_MOESM1_ESM.docx]

Supplementary information

**Investigation on the applicability of a long-range reverse-transcription quantitative polymerase chain reaction assay for the rapid detection of active viruses**

Masato Yasuura^1,^*^, †^, Yuki Nakaya^1, 2, †^, Hiroki Ashiba^a^, and Takashi Fukuda^a^

^1^Sensing System Research Center, National Institute of Advanced Industrial Science and Technology (AIST), Central 5, 1-1-1 Higashi, Tsukuba, Ibaraki 305-8565, Japan

^†^ These authors contributed equally to this work

^2^Division of Virology, Department of Infection and Immunity, School of Medicine, Jichi Medical University, 3311-1 Yakushiji, Shimotsuke, Tochigi 329-0498, Japan

*To whom correspondence should be addressed:

Sensing System Research Center, National Institute of Advanced Industrial Science and Technology (AIST), Central 5, 1-1-1 Higashi, Tsukuba, Ibaraki 305-8565, Japan

Email: yasuura-masato@aist.go.jp, Phone: +81-29-861-3679

*Conventional RT-qPCR*

The conventional RT-qPCR measurements were carried out to be compared with the LR-RT-qPCR according to the published studies with minor modifications [18]. Different from the assays in the main text, another lot of the same strain IAV stock (A/Panama/2007/1999 [H3N2], 8.75 log [TCID_50_/0.1 mL]) was used in the measurements. The IAV stock was diluted by PBS (-) to the same titers as the averages of the non-treated samples in Figure 1 (5.25 log [TCID_50_/0.1 mL]) for use. The probe and primer sequences were derived from the IAV detection protocols released by the World Health Organization (WHO) [26]. The isolation of viral RNA was performed in the same way described in the 'Long-range RT-qPCR' section. The isolated RNA was subjected to RT-qPCR with the TaqMan Fast Virus 1-Step Master Mix (Thermo Fisher Scientific K.K., Tokyo, Japan), a TaqMan probe (5’-FAM-ATY­TCGG­CTTTGAGGGGGCCTG-MGB-3’), and a primer pair (Forward: 5’-CCMAGG­TCGAAACGTAYGTTCTCTC­TATC-3’, Reverse: 5’-TGACAGRATYGGTCTTGTCTTTAGCCAY­TCCA-3’). Thermal cycling was performed on the LightCycler 96 (Roche Diagnostics K.K., Tokyo, Japan) for reverse transcription at 50°C for 5 min, denaturation of the RT polymerase at 95°C for 20 s, and 40 cycles of PCR at 95°C for 3 s and 60°C for 30 s. Copy number quantification was carried out with the simultaneous measurement of the 10-fold serially diluted standard DNA. An assay was performed in a duplicate experiment. The LoD of the conventional RT-qPCR assay was 0.203 copies/μL. Each copy number was divided by the copy number of non-treated IAV to calculate the copy number ratio. Data represent the three independent experiments and the mean ± standard error of them.

Supplementary Figure 1 shows additional data on the conventional RT-qPCR. The data on the LR-RT-qPCR and titer is the same as in Figure 1. In this experiment, the copy numbers on the conventional RT-qPCR measurements were not influenced by the heat-inactivation. Therefore, the LR-RT-qPCR assay is more suitable than the conventional RT-qPCR assay to monitor infectious risk at the site where there may be remnants of heat-inactivated IAVs.


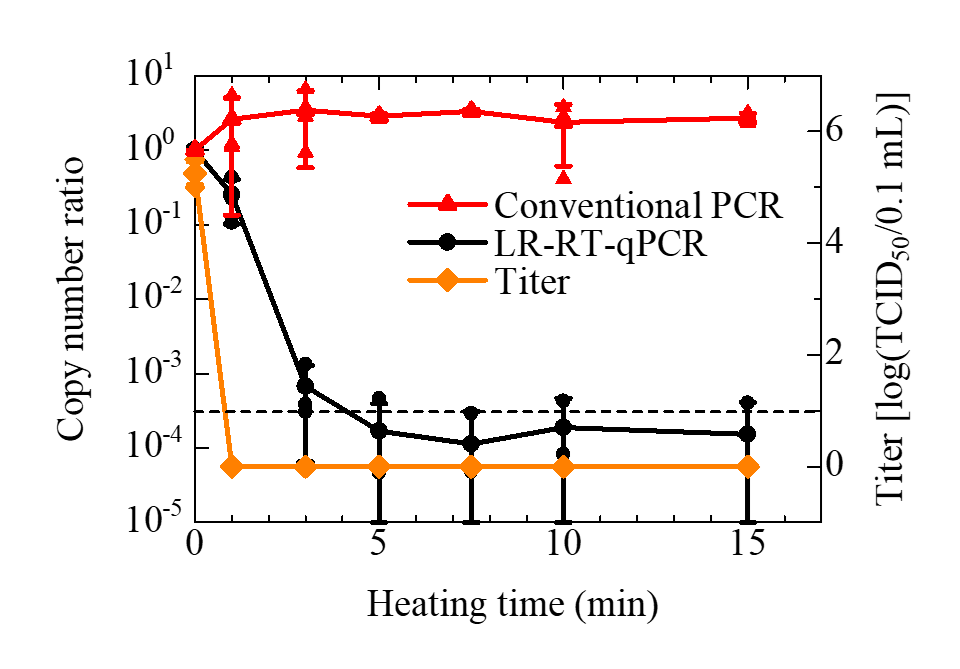


Supplementary Figure 1. Effect of 100 °C heating on IAV infectivity and copy numbers. IAV suspensions were heated with a block incubator for increasing periods of time between 1 and 15 min. The data on the LR-RT-qPCR and titer is the same as in Figure 1. The ratio of copy numbers obtained using the conventional RT-qPCR is also plotted on the graph (▲). The conventional RT-qPCR assay was carried out independently three times with duplicate wells per sample and plots represent the mean ± standard error and individual values. A broken line indicated the LoD of LR-RT-qPCR. The data set of conventional PCR were indicated in Additional Table 6 (See ‘Additional Tables’).

*Viral samples*

The viral samples including Panama and PR8 strains were propagated in MDCK cells at our laboratory. The aliquots were not available because the cultivation lot in this report is exhausted.
